# Application of a modified gaseous exposure system to the *in vitro* toxicological assessment of tobacco smoke toxicants

**DOI:** 10.1002/em.21876

**Published:** 2014-06-02

**Authors:** Damien Breheny, Fiona Cunningham, Joanne Kilford, Rebecca Payne, Deborah Dillon, Clive Meredith

**Affiliations:** ^1^ British American Tobacco Group R&D, Southampton Hampshire SO15 8TL United Kingdom; ^2^ Covance Laboratories Ltd Otley Road, Harrogate North Yorkshire HG3 1PY United Kingdom

**Keywords:** ethylene oxide, Ames, gaseous exposure, *in vitro*, Vitrocell^®^ VC 10

## Abstract

Tobacco smoke is a complex mixture of over 6,000 individual chemical constituents. Approximately 150 of these have been identified as ‘tobacco smoke toxicants’ due to their known toxicological effects. A number of these toxicants are present in the gaseous phase of tobacco smoke. This presents a technical challenge when assessing the toxicological effects of these chemicals *in vitro*. We have adapted a commercially available tobacco smoke exposure system to enable the assessment of the contribution of individual smoke toxicants to the overall toxicological effects of whole mainstream cigarette smoke (WS). Here we present a description of the exposure system and the methodology used. We use the example of a gaseous tobacco smoke toxicant, ethylene oxide (EtO), a Group 1 IARC carcinogen and known mutagen, to illustrate how this methodology can be applied to the assessment of genotoxicity of gaseous chemicals in the context of WS. In the present study we found that EtO was positive in *Salmonella typhimurium* strain YG1042, a strain that is sensitive to tobacco smoke. However, EtO did not increase the mutagenicity of the WS mixture when it was added at greatly higher concentrations than those found typically in WS. The findings presented here demonstrate the suitability of this exposure system for the assessment of the mutagenic potential of gases *in vitro*. Whilst we have focused on tobacco smoke toxicants, this system has broad application potential in studying the biological effects of exposure to a wide range of gaseous compounds that are present within complex aerosol mixtures. Environ. Mol. Mutagen. 55:662–672, 2014. © 2014 The Authors. Environmental and Molecular Mutagenesis published by Wiley Periodicals, Inc., on behalf of Environmental Mutagen Society

## INTRODUCTION

The toxicological assessment of aerosols and gases presents a technical challenge that has been the subject of intense research over the years. In parallel with the development of *in vivo* inhalation methods [Coggins, 2010; Owen, 2013], there has also been significant progress made in recent years in approaches to expose cells *in vitro* to chemicals and complex mixtures that pose a toxicological risk via the inhalation route [Aufderheide et al., 2002; Aufderheide, 2005; Bakand et al., 2005]. These toxicants can be derived from a wide variety of sources, and humans can be exposed as a consequence of their occupation, environment or lifestyle. For example, the oral and respiratory epithelia of smokers are directly exposed to a complex mixture of chemicals, many of which are known to be toxicants that induce local and/or systemic effects [Brody and Steiling, 2011; Huertas and Palange, 2011]. A number of *in vivo* exposure systems have been developed to facilitate the study of the effects of the whole smoke mixture on both mammalian and bacterial cells, and this has been the subject of a recent review [Thorne and Adamson, 2013].

In addition to studies on the biological responses of cells to the whole smoke mixture, there is increasing interest in understanding the relative contributions of individual tobacco smoke toxicants to smoking related disease. The causal link between smoking and a number of diseases has been long established [Doll et al., 2004], and it has been reported that there are over 6,000 chemicals present in mainstream tobacco smoke [Rodgman and Perfetti, 2013]. However, relatively little is yet known about the mechanisms by which tobacco smoke causes disease, and which subset of the multitude of chemicals present within smoke are responsible. In recent years the World Health Organization Study Group on Tobacco Product Regulation (TobReg) has proposed a list of 18 toxicants for mandatory monitoring or lowering in cigarette smoke, based on their involvement in diseases that result from smoking [Burns et al., 2008]. In March 2012, the Food and Drug Administration (FDA) in the USA established a list of 93 Harmful and Potentially Harmful Constituents (HPHC) in tobacco products and tobacco smoke [Food and Drug Administration, 2012]. This nonexhaustive list includes constituents that have previously been identified by other national and international bodies as being harmful or potentially harmful, and the FDA will require that tobacco companies in the USA report the levels of each of these in all of their cigarette products. Since reliable and robust analytical techniques have not been established for all 93 constituents, a subset of 20 HPHCs has been initially proposed for reporting. Further insight into the roles of individual tobacco smoke toxicants in smoking related disease will aid in prioritizing the reduction in levels of toxicants in smoke in the interest of harm reduction.

A number of the toxicants in tobacco smoke are present in the gaseous phase of this complex mixture. Due to the technical difficulties associated with the testing of gaseous chemicals, the biological effects of many of these individual smoke toxicants have not been widely studied *in vitro* to date. We have previously characterized a Vitrocell^®^ VC 10 whole smoke exposure system that has been used to evaluate the cytotoxic and mutagenic effects of mainstream tobacco smoke on mammalian and bacterial cells, respectively [Thorne et al., 2013]. The VC 10 is a single syringe, rotary style smoking machine that typically transfers mainstream tobacco smoke into a continuous flow dilution system. Smoke is diluted under turbulent conditions produced by the introduction of the diluting air in a perpendicular direction to the stream of smoke. The final concentration of smoke that is delivered to the cell system of interest can be adjusted by varying the diluting airflow rate. A vacuum is used to divert the diluted smoke from the dilution system into exposure modules, which can house an Ames plate or a mammalian cell culture insert. In this way individual cultures can be exposed independently to diluted tobacco smoke or other aerosols.

The purpose of the present study was to adapt the Vitrocell^®^ system to enable the exposure of cell systems to gaseous toxicants in combination with WS exposure in a set of spiking experiments. This approach would facilitate the assessment of the relative contributions of individual gaseous constituents of WS to the overall toxicological effects of this complex mixture. Ethylene oxide (EtO) was used as a test gas for the development and application of this system, as it is a well‐known genotoxic agent both *in vitro* and *in vivo*, as well as being a carcinogen in laboratory animals [Victorin and Stahlberg, 1988; Thier and Bolt, 2000; IARC, 2008]. EtO is an important raw material used in the production of mono‐ethylene glycol, which is globally produced on a massive scale. It is also used as a fumigant and sterilizing agent, and is present in tobacco smoke, which is thought to be the only significant non‐occupational source of human exposure to EtO [IARC, 2008]. It has previously been shown to be mutagenic in the Ames assay in *Salmonella typhimurium* strains TA100 and TA1535 [Victorin and Stahlberg, 1988; Agurell et al., 1991]. In this study we assessed the mutagenic potential of EtO (on its own and spiked into WS) in *Salmonella typhimurium* strain YG1042, a strain derived from TA100 that has previously been shown to be sensitive to tobacco smoke [Aufderheide and Gressmann, 2008; Kilford et al., 2014].

Whilst this paper describes the development and application of a novel method for exposing bacterial cells to a gaseous tobacco smoke toxicant, both alone and spiked into tobacco smoke, the methodology could equally be applied to testing of any gaseous compound of interest in this way in a number of *in vitro* cellular systems or *ex vivo* tissues.

## MATERIALS AND METHODS

### Chemicals and Reagents

All chemicals and reagents were obtained from Sigma‐Aldrich (Gillingham, UK) unless otherwise stated.

EtO gas was purchased as a 1.5% (15,000 ppm) mixture in nitrogen from BOC Industrial Gases UK. Certified concentration values provided with each of the three 1.5% EtO cylinders used in this study ranged from 1.47 to 1.50%.

### Reference Cigarettes

3R4F reference cigarettes were obtained from the University of Kentucky, Kentucky, USA. Prior to smoking, cigarettes were conditioned for at least 48 hr and no more than 10 days at 22 ± 1°C and 60 ± 3% relative humidity according to International Organization of Standardization (ISO) 3402:1999.

### Exposure *S*etup

Experiments were performed on the Vitrocell^®^ VC 10 Smoking Robot (Vitrocell^®^ Systems, Waldkirch, Germany) using a Vitrocell^®^ valve spiking setup which was operated by the VC 10 Smoking Robot control software (Vitrocell^®^ Systems, Waldkirch, Germany). Two different experimental setups were used during this study. While it was possible to attach up to four dilution bars to the VC 10 used in the first set of experiments (serial number VC10/221211), the initial version of the Smoking Robot control software (PLC version 1.4) could only operate one set of spiking valves at a time. Therefore, only one dilution bar could be employed and, as a result, only one concentration of test gas, or test gas/WS mixture could be assessed in each experimental run. As we developed the methodology further, a newly modified VC 10 (serial number VC10/300412) was introduced for the later experiments. This VC 10 was accompanied by an updated version of the control software (PLC version 1.6) which facilitated the simultaneous operation of four sets of valves to direct gas to four dilution bars, each set to a different diluting airflow. Thus, up to four concentrations of test gas or test gas/WS mixture could be evaluated within the same experimental run.

**Figure 1:**
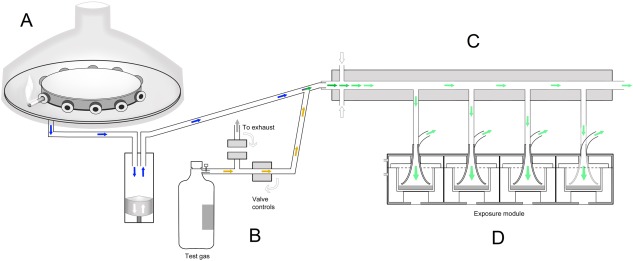
Schematic cross‐section of the Vitrocell® VC 10 whole mainstream smoke (WS) and gaseous exposure system. The setup consists of a VC 10 Smoking Robot (A) and a separate but interconnected system for exposing cells to a test gas (B). This setup is used to expose bacterial cells to WS (blue arrows) and to test gas (EtO; yellow arrows), either individually or as a spiked mixture (green arrows). The desired final concentration of the test agent is achieved by manipulation of the flow rate of the diluting air which enters the dilution bar (C) at a perpendicular angle to the flow of the test agent. For the WS spiking experiments the concentration of EtO in WS is determined by adjusting the flow rate of the gas entering the stream of WS. This spiked mixture then enters the dilution bar, where the final concentration is achieved by altering the diluting air flow rates. Dilution and transit of test agent occur within the dilution bar via turbulent mixing. Ames plates are exposed to the test agent in a Vitrocell^®^ AMES 4 stainless steel module (D) which docks under the dilution system. A vacuum is applied to the module which draws the diluted test agent from the dilution bar, into the module via the ‘trumpet’ inlets and out of the module to exhaust. A vacuum rate of 5 mL/min/well was used in all experiments. Due to the continuous diluting airflow, test agent remaining in the dilution system transits to exhaust away from the module (modified from [Adamson et al., 2013]).

For the purposes of the present study, the Vitrocell^®^ VC 10 WS exposure system was connected to a separate gaseous exposure system to allow either separate or simultaneous exposure to WS and test gas (Fig. 1). The test gas was contained in a cylinder, and its flow was controlled by a system of valves and mass flow controllers (Fig. 1B). For spiking experiments, the test gas was introduced into the freshly‐generated WS immediately prior to entering the dilution bar, where the spiked WS was further mixed and diluted to the desired concentrations using air (Fig. 1C). The VC 10 controlled the opening and closing of the Vitrocell^®^ valve system such that gas was released to coincide with each puff of smoke.

### Health and *S*afety *C*onsiderations

The VC 10 and gas cylinders were located in an extraction chamber which was maintained under constant negative pressure with approximately 40 full air replacements per minute (Controlled Air Solutions, Heywood, Lancashire, UK). All extracts were passed through Pre, HEPA, and carbon filters before being released via a high velocity discharge stack. The VC 10 and Vitrocell^®^ valve system used to control gas and smoke flow were operated via a control panel and computer external to the chamber. Air quality within the chamber was monitored continuously using AreaRAE Steel monitors, (RAE Systems, Kastrup, Denmark) viewed remotely using ProRAE Remote software version 3.05.

### Ames *A*ssay

*Salmonella typhimurium* (strain YG1042) was used in the Ames assay and was obtained from the National Institute of Health Science (Tokyo, Japan). The bacterial strain YG1042 is a derivative of strain TA100 with a histidine base‐pair substitution [Hagiwara et al., 1993]. It carries an additional plasmid (pYG233) encoding for overexpression of nitroreductase and *O*‐acetyltransferase genes. Strain characteristic assessments were carried out according to previous reported methodologies [de Serres and Shelby, 1979; Maron and Ames, 1983]. Bacteria were cultured at 37°C for 8 hr in nutrient broth, containing ampicillin (25 µg/mL) and kanamycin (25 µg/mL) to obtain cells in the late log phase of growth.

In the standard plate‐incorporation method, bacteria are embedded in a top agar with the test material. However, some gases may not penetrate the top agar and therefore a method whereby the bacteria are fully exposed may be more appropriate. The spread‐culture method [Araki et al., 1994; Aufderheide and Gressmann, 2007] was therefore selected for this study. Approximately 2 × 10^7^ bacterial cells were plated on to 35 mm Vogel–Bonner E agar plates in 75 µL sodium phosphate buffer (pH 7.4, containing 48.8 µg/mL biotin and 40 µg/mL histidine) using a spread plate methodology, with a total plating volume of 95 µL. WS and spiked WS exposures were performed in the presence and absence of an exogenous metabolic activation system (Aroclor 1254‐induced rat liver S9 from male Sprague‐Dawley rats (MolTox^®^, Molecular Toxicology, USA). Where S9 was used, it was incorporated at 10% in a buffer containing cofactors as previously described [Ames et al., 1975]. Plates were then transferred to an anhydrous incubator set at 37°C until dry.

### EtO Exposure

The EtO gas flow rate was set to 0.263 L/min, which is equivalent to smoke exiting the piston of a VC 10 Smoking Robot during an ISO 3308:2000 smoking regime when using an 8‐sec exhaust. Gas flow to the modules and to exhaust was controlled by the Vitrocell^®^ valve system. The valves were controlled via the VC 10 software which was programmed such that EtO gas was supplied to the test system for 8 sec of every 60 sec, and directed to exhaust for the remaining 52 sec. EtO was diluted into a constant flow of compressed air through the Vitrocell^®^ dilution system. Different concentrations of EtO were achieved by varying the flow rate of diluting air, resulting in a range of four concentrations from approximately 1,000 to approximately 6,000 ppm (Table 1). The top concentration (5,950 ppm) was determined on the basis that it was the highest concentration achievable with a 1.5% EtO gas cylinder when the minimum diluting air flow rate was set at 0.4 L/min. This diluting air flow rate was selected to be higher than the gas flow rate to encourage thorough mixing in the dilution bar. A 1.5% EtO cylinder was selected to allow us to achieve a similar concentration range to one that had previously been shown to induce micronuclei in V79 cells following a 30 min exposure [Zhong et al., 1991]. All experiments were performed using a 24 min exposure period (the equivalent of a 3 cigarette exposure smoked at 8 puffs per cigarette according to ISO 3308:2000 guidelines) with a 5 mL/min/well vacuum. The VC 10 was modified during the course of these studies and later experiments were performed on the VC 10 with updated software. Details of the individual VC 10 setups used in each set of experiments are described in the “Results”.

**Table 1:** Diluting Air Flow Rates Used To Generate Final Testing Concentrations of EtO

Cylinder concentration (ppm)	Gas flow rate (L/min)	Diluting air flow rate (L/min)	Final concentration (ppm)
15,000	0.263	3.7	995
		1.2	2,697
		0.6	4,571
		0.4	5,950

### WS Exposure

YG1042 cells were exposed to various concentrations of WS for 24 min (3 cigarettes) according to the ISO smoking regime with an 8‐sec exhaust, both in the presence and absence of S9, using the methodology previously described [Kilford et al., 2014]. Different concentrations of WS were obtained using diluting air flow rates of 1.0, 4.0, 8.0, and 12.0 L/min; the same as those tested previously in our laboratory. WS exposures do not utilize the valve spiking system and therefore it was possible to test all WS concentrations at the same time.

### EtO‐Spiked WS Exposure

Spiking of WS with different concentrations of EtO was achieved in two steps. The EtO was first introduced at varying flow rates into WS. Smoke generation was performed according to the ISO smoking regime, with an 8 sec exhaust, and thus smoke traveling from the smoking machine was assumed to be at a flow rate of 0.263 L/min. The remaining dilution of the spiked mixture was achieved in a second step, where diluting air entering the dilution bar, perpendicular to the flow of the spiked mixture, was used to adjust the total dilution flow rate up to either 1 or 4 L/min.

Table 2 shows details of how final concentrations of EtO in WS were achieved for experiments performed in both the presence and absence of S9. WS spiking experiments were performed using the initial VC 10 setup, where a single treatment concentration was tested in each experimental run.

**Table 2:** Details of EtO‐Spiked WS Experiments

S9	Cylinder concentration (ppm)	Gas flow rate (L/min)	Diluting air flow rate (L/min)	Final EtO concentration in smoke (ppm)^a^	Final concentration to cells (ppm)
+	15,000	0.30^b^	3.70	7,993	1,056
		0.45^c^	0.55	9,467	5,344
		0.55^b^	3.45	10,148	1,935
		0.65^c^	0.35	10,679	7,720
		0.80	0.20	11,289	9,501
‐	15,000	0.10	2.90	4,132	460
		0.60	2.40	10,428	2,758
		1.10	1.90	12,106	5,057

a Prior to mixing in the dilution bar.

b Treatment concentrations included in Experiments 2 and 3 only.

c Treatments concentrations included in Experiment 1 only.

In the initial experimental VC 10 setup for EtO exposure the diluting air flow rate and vacuum were set and checked using mass flow meters (Analyt‐MTC GmbH, Mulheim, Germany). The gas flow was set and controlled by a mass flow controller. In the revised setup used for EtO exposure in later experiments, and for the WS and WS‐EtO experiments, the vacuum was still set with mass flow meters but gas and diluting air were controlled by mass flow controllers. Air controls, using an air flow rate of 0.2 L/min, untreated negative controls, as well as positive control treatments with sodium azide (NaN_3_; −S9 control) or 2‐aminoanthracene (AAN; +S9 control), were included in each experiment. Untreated and positive controls were maintained at room temperature for the duration of the exposure. Following exposure, the system was purged for approximately 20 min with air to remove all residual test gas/aerosol before retrieval of the agar plates. This included a 15‐min purge cycle using the VC10 to run the diluting air through the dilution system plus a 6‐min purge using air from a second cylinder to purge the remaining tubing.The plates were then incubated at 37°C for 3 days before being examined for signs of toxicity and scored as described below.

### Colony Scoring and Acceptance Criteria

Plates were assessed for any evidence of toxicity, which is characterized by thinning of the background bacterial lawn and may be accompanied by a decrease in the number of revertant colonies. Plates were then scored using a Sorcerer Image Analyzer (Perceptive Instruments, Haverhill, UK). As the present experiments were performed using 35 mm plates for which sufficient historical control data was not available, the data from the vehicle and positive control plates were compared against data ranges for TA100 (the parent strain) in the standard regulatory Ames assay (on 85 mm plates) held at Covance Laboratories, which were first scaled down to give equivalent colony counts on 35 mm plates. These TA100 historical data from 85 mm plates are based on a large number of experiments and are consistent with accepted spontaneous revertant ranges [de Serres and Shelby, 1979]. An observed range of spontaneous revertant numbers for treatments performed with the scaled down spread‐plate methodology in YG1042 had also previously been generated by Covance Laboratories. These YG1042 data were comparable to the scaled down historical control data for TA100. Experiments were considered acceptable if the untreated, air and positive control mean revertant numbers were comparable with both the observed ranges for YG1042 and the scaled‐down historical control ranges for TA100.

### Data Presentation and Statistics

Mutagenicity determination was achieved by the use of the Dunnett's test, combined with a prerequisite for a minimal fold increase in revertant numbers. The test agent was deemed to induce a positive mutagenic response if it produced a reproducible, concentration‐related increase in revertant numbers to levels that were at least twofold the concurrent air control. In addition, the increase should be significant at the 1% level (*P* ≤ 0.01) using Dunnett's test.

## RESULTS

### EtO Exposure

**Figure 2:**
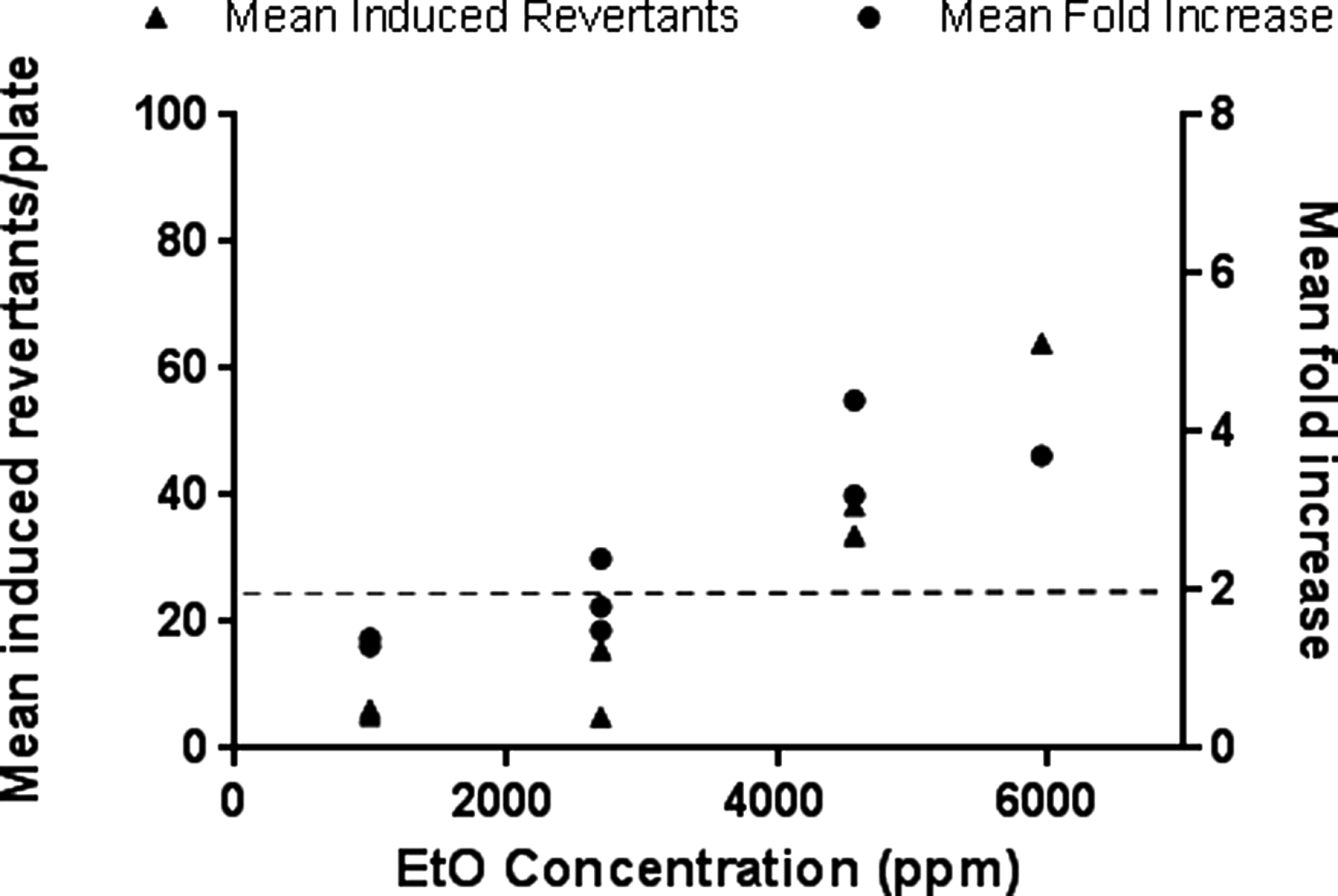
Mutagenicity of EtO in *Salmonella typhimurium* strain YG1042. Bacteria were exposed to EtO at 3 concentrations per experiment (995, 2,697, and 5,950 ppm in Experiment 1; 995, 2,697, and 5,950 ppm in Experiments 2 and 3) over a 24‐min period, under conditions that simulated the ISO smoking regime using an 8‐sec exhaust. Each concentration of EtO was tested separately, and the mean fold increase in revertant number was calculated against the concurrent air control. This was repeated on three occasions to obtain three datasets. Following the results from the first experiment, the top concentration was lowered to 4,571 ppm for the subsequent two experiments. Data from the three datasets are presented as mean‐induced revertant counts (calculated by subtracting the background air control mean revertant number from the treatment group mean revertant number), and as mean fold increases compared to the concurrent air control. A ≥2‐fold mean fold increase indicates a positive mutagenic response (as indicated by the dashed line).

Initial experiments were performed to establish the concentrations of EtO required to elicit a mutagenic response in YG1042 under treatment conditions previously established for whole smoke exposure using the VC 10 [Thorne et al., 2013]. The data obtained from the testing of EtO using our initial experimental system are shown in Figure 2. The mean revertant numbers for the positive, air and untreated controls were comparable to the control ranges. As each concentration of EtO was tested in a separate exposure, the fold increase in mean revertant number at each concentration was calculated from the concurrent air control. Each datapoint was calculated from three replicate Ames plates. Following the first set of experiments, it was noted that the number of revertants in each of the three plates varied considerably at the top concentration (5,950 ppm). This could be attributed to inadequate mixing of EtO and air at the low dilution flow rate (0.4 L/min) used to generate this final concentration. As a consequence, the lowest diluting airflow rate was increased to 0.6 L/min, resulting in a top concentration of 4,571 ppm EtO in the following two sets of experiments. The lower two concentrations were kept the same, as they gave a slightly elevated response above the untreated control in the first experiment. The third replicate set of experiments was performed on a modified VC 10 with revised software that allowed the use of all four dilution bars and therefore simultaneous exposure of four treatment groups to EtO. For the purposes of repeating the earlier experiments, the new VC 10 was setup to mimic the previous exposure system and individual experimental runs were performed for each concentration of EtO. The results were comparable to the previous two sets of experiments. EtO was shown to induce revertants in a concentration‐dependent manner, while there was no evidence of toxicity observed at any of the concentrations. While there was a mean fold increase in revertants of 3.7 at 5,950 ppm, this was not significant in Dunnett's test due to the high level of variability between replicate values. 4,571 ppm EtO induced a ≥2‐fold increase in mean revertant numbers, accompanied by a statistically significant difference from air controls in Dunnett's test in the following two experiments, albeit at the 5% level in one experiment.

**Figure 3:**
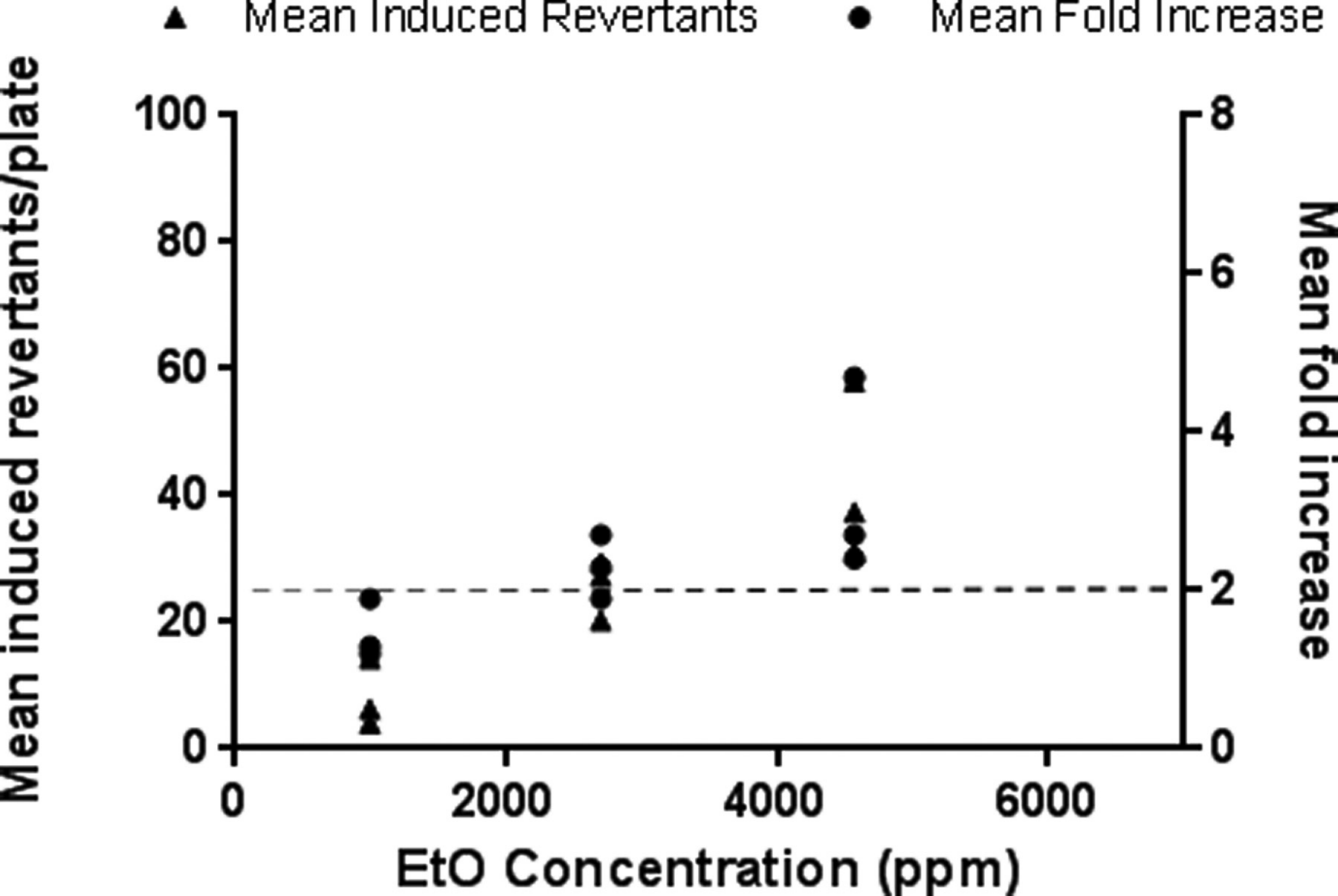
Mutagenicity of EtO in *Salmonella typhimurium* strain YG1042. Bacteria were exposed to EtO at concentrations of 995, 2,697, and 4,571 ppm over a 24‐min period, under conditions that simulated the ISO smoking regime. In each experiment, all three concentrations of EtO were tested simultaneously, and the mean fold increase in revertant number was calculated against the concurrent air control for that experiment. A ≥2‐fold mean fold increase indicates a positive mutagenic response (as indicated by the dashed line). Data from three experiments are presented as mean induced revertant counts (calculated by subtracting the background air control mean revertant number from the treatment group mean revertant number), and as mean fold increases compared to air control.

Whilst the original system setup was successful in producing data that were both reproducible and in accordance with our acceptance criteria, this was not optimal from either a pragmatic or a scientific perspective. The modified VC 10 greatly increased the amount of data that could be generated from a single test run, and meant that data from all concentrations tested within that run could be assessed against the same air control treatment group. This new setup was assessed, again using EtO as the test gas. EtO was tested at concentrations of 995, 2,697, and 4,571 ppm in each of three experiments performed. This was to allow direct comparison with the same three concentrations that gave a positive, concentration‐related response to EtO using the initial exposure system setup. The results of the three experiments are shown in Figure 3. There was no evidence of toxicity observed at any of the concentrations tested. A clear concentration‐related increase in both mean revertant number and mean fold change was observed. In addition, the values obtained for both of these parameters were very similar to those found using the original single exposure setup. Treatment with 4,571 ppm EtO resulted in a mean fold increase of ≥2 in all three experiments, and this was significant at the 1% level in Dunnett's test in two of those experiments and at the 5% level in the other experiment. Data from all experiments are summarized in Tables 3 and 4.

**Table 3:** Summary of Data Obtained from EtO Treatment Using Original Exposure Setup

Experiment	Treatment (ppm)	Mean revertants	Standard deviation	Fold increase	Dunnett's test result
1	Air	21.0	6.1		
	995	26.3	6.7	1.3	NS
	Air	29.7	8.5		
	2,697	45.3	15.1	1.5	NS
	Air	23.7	6.5		
	5,950	87.7	48.7	3.7	NS
				
2	Air	16.5	7.1		
	995	22.7	1.5	1.4	NS
	Air	11.3	1.2		
	2,697	27.0	6.1	2.4	b
	Air	15.3	3.2		
	4,571	49.0	18.1	3.2	a
				
3	Air	15.3	4.0		
	995	21.3	5.7	1.4	NS
	Air	18.3	2.1		
	2,697	33.3	5.1	1.8	b
	Air	11.3	5.1		
	4,571	49.7	17.7	4.4	b

NS = not significant

a Significant (*P* ≤ 0.05).

b Significant (*P* ≤ 0.01).

**Table 4:** Summary of Data Obtained from EtO Treatment Using Revised Exposure Group

Experiment	Treatment (ppm)	Mean revertants	Standard deviation	Fold increase	Dunnett's test result
1	Air	22.3	1.2		
	995	26.3	2.1	1.2	NS
	2,697	51.7	7.4	2.3	b
	4,571	59.7	8.4	2.7	b
				
2	Air	15.7	1.5		
	995	30.0	5.2	1.9	NS
	2,697	43.0	12.8	2.7	a
	4,571	73.7	16.0	4.7	b
				
3	Air	22.7	17.6		
	995	29.0	9.6	1.3	NS
	2,697	43.0	17.1	1.9	NS
	4,571	53.7	10.6	2.4	a

NS = not significant.

a Significant (*P* ≤ 0.05).

b Significant (*P* ≤ 0.01).

### Spiking of WS with EtO

#### WS Exposure

**Figure 4:**
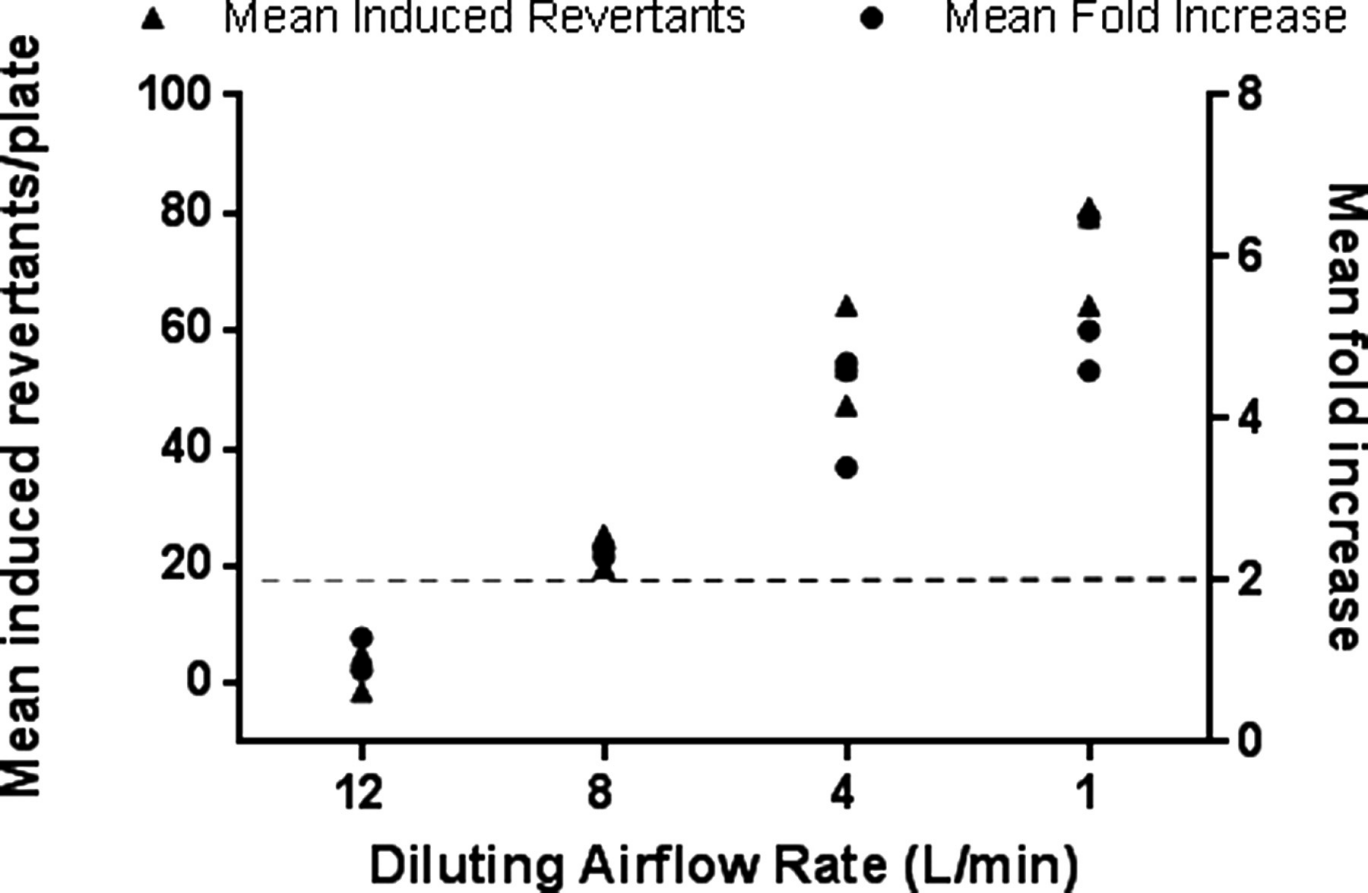
Mutagenicity of WS in *Salmonella typhimurium* strain YG1042 in the presence of S9. Bacteria were exposed to WS diluted in air using airflow rates of 1, 4, 8, and 12 L/min over a 24‐min period, following the ISO smoking regime with an 8‐s exhaust. All four concentrations of WS were tested at the same time, and the mean fold increases in revertant number were calculated against the concurrent air control. This was repeated on three occasions to obtain three datasets. Data from the three datasets are presented as induced revertant counts (calculated by subtracting the background air control mean revertant number from the treatment group mean revertant number), and as mean fold increases compared to the air control. A ≥2‐fold mean fold increase indicates a positive mutagenic response (as indicated by the dashed line).

**Figure 5:**
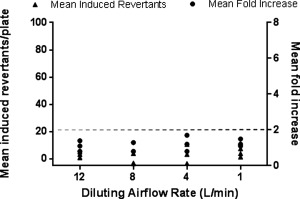
Mutagenicity of WS in *Salmonella typhimurium* strain YG1042 in the absence of S9. Bacteria were exposed to WS diluted in air using airflow rates of 1, 4, 8, and 12 L/min over a 24‐min period, following the ISO smoking regime with an 8‐sec exhaust. All four concentrations of WS were tested at the same time, and the mean fold increases in revertant number were calculated against the concurrent air control. This was repeated on three occasions to obtain three datasets. Data from the three datasets are presented as mean induced revertant counts (calculated by subtracting the background air control mean revertant number from the treatment group mean revertant number), and as mean fold increases compared to the air control. A ≥2‐fold mean fold increase indicates a positive mutagenic response (as indicated by the dashed line).

Before performing the EtO spiking experiments it was necessary to establish the baseline response of YG1042 to WS using the same VC 10. In the presence of S9, WS exposure for 24 min under ISO conditions resulted in a concentration‐related increase in mean revertant numbers and a corresponding increase in fold change over the control (air) treatment group (Fig. 4). At airflow rates of ≤8 L/min, this increase in response was twofold or greater. This contrasted with a lack of response in the same test system when S9 was omitted (Fig. 5). These findings were comparable to those previously demonstrated in the same laboratory using a different VC 10 smoking engine [Kilford et al. 2014].

#### EtO‐Spiked WS Exposure

**Figure 6:**
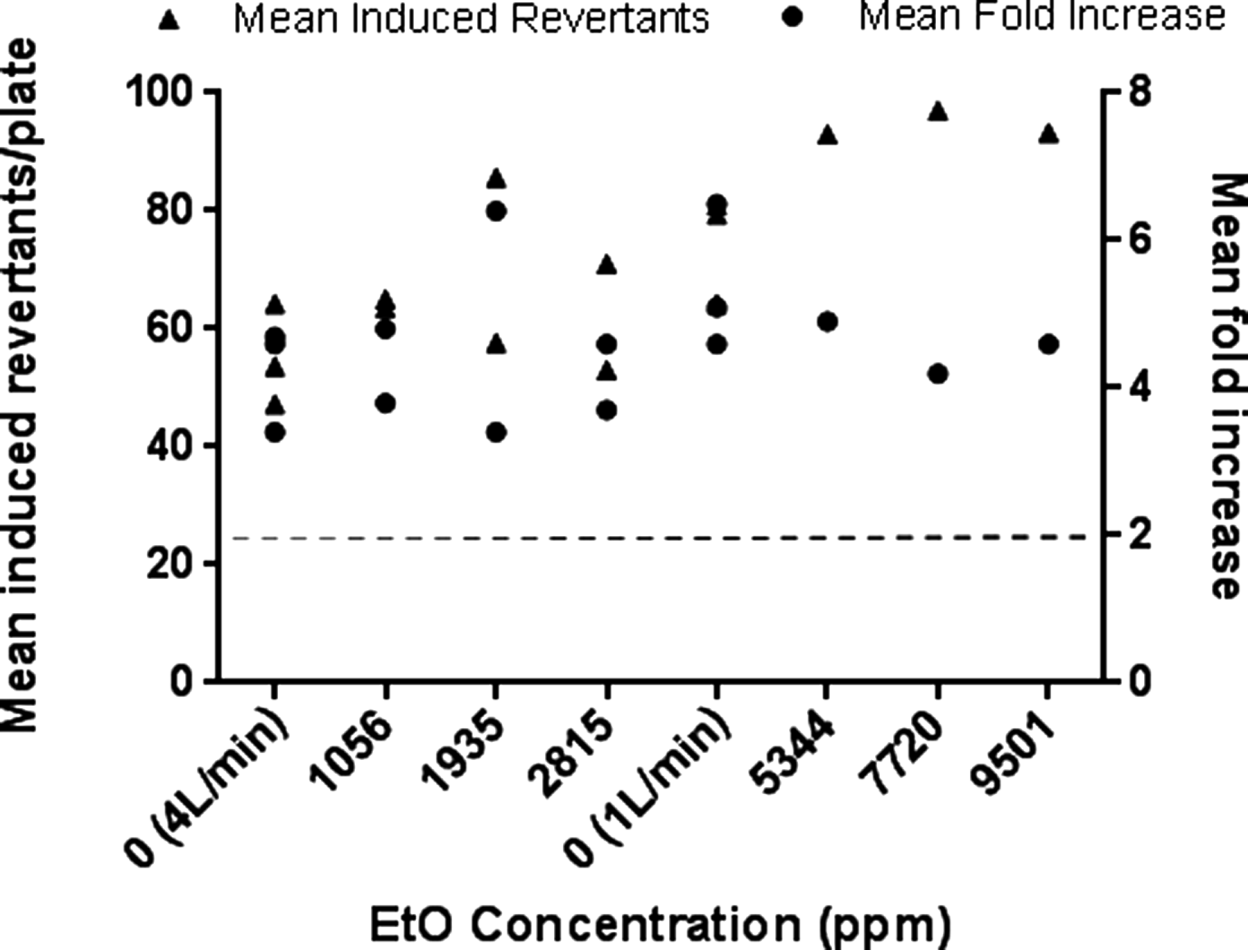
Mutagenicity of EtO‐spiked WS in *Salmonella typhimurium* strain YG1042 in the presence of S9. A 35 mL puff of smoke, exhausted over 8 sec (equivalent to a flow rate of 0.263 L/min) was spiked with EtO. Bacteria were exposed to EtO‐spiked WS diluted in air using total diluting flow rates (gas flow + diluting air flow) of 1 L/min (resulting in final EtO concentrations of 5,344, 7,720, and 9,501 ppm (*n* = 1)) and 4 L/min [resulting in final EtO concentrations of 1,056, 1,935, and 2,815 ppm (*n* = 2)] over a 24‐min period. Each concentration of EtO‐spiked WS was tested separately, and the mean fold increase in revertant number was calculated against the concurrent air control. Data from the three datasets are presented as mean induced revertant counts (calculated by subtracting the background air control mean revertant number from the treatment group mean revertant number), and as mean fold changes compared to the air control. A ≥2‐fold mean fold increase indicates a positive mutagenic response (as indicated by the dashed line). Unspiked WS control data for both the 1 and 4 L/min dilutions from Fig. 4 are included in the graph to allow direct comparison with corresponding EtO‐spiked WS data.

Cells were exposed to EtO‐spiked WS at final concentrations of 5,344, 7,720, and 9,501 ppm in an initial experiment in the presence of S9. This was achieved using a total diluting flow rate (gas flow rate + diluting airflow rate) of 1 L/min (see Table 2). These concentrations were selected after a preliminary range finder experiment performed with a 1% EtO cylinder at lower concentrations (up to 6,334 ppm and therefore comparable to what the cells received when treating with EtO only) had no effect on the WS response (data not shown). The 1 L/min diluting airflow rate was selected based on WS exposure data, where it was used to produce the highest concentration of WS tested, and a mean fold increase in revertants of between 4.6 and 6.5 (see Fig. 4). To investigate whether EtO would affect the mutagenicity of WS at lower concentrations of WS, two further experiments were performed using a higher total diluting airflow rate of 4 L/min (also used in the WS experiments, where it gave a mean fold increase in revertants of between 3.4 and 4.6). This resulted in lower final concentrations of EtO in WS of 1,056, 1,935, and 2,815 ppm. The results of these spiking experiments are presented in Figure 6, where data from the WS exposures at equivalent flow rates (1 and 4 L/min) are also included for comparison purposes. The mean fold increases in the EtO‐spiked WS treatment groups were comparable to those observed with WS treatment alone previously, indicating that EtO did not significantly add to the mutagenic potential of WS at any of the concentrations tested.

**Figure 7:**
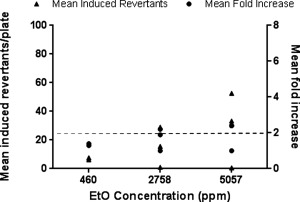
Mutagenicity of EtO‐spiked WS in *Salmonella typhimurium* strain YG1042 in the absence of S9. A 35 mL puff of smoke, exhausted over 8 sec (equivalent to a flow rate of 0.263 L/min) was spiked with EtO. Bacteria were exposed to EtO‐spiked WS diluted in air using a total diluting flow rate (gas flow + diluting air flow) of 3 L/min (resulting in final EtO concentrations of 460, 2,758, and 5,057 ppm) over a 24 min period. Each concentration of EtO‐spiked WS was tested separately, and the mean fold increase in revertant number was calculated against the concurrent air control. Data from the three datasets are presented as mean induced revertant counts (calculated by subtracting the background air control mean revertant number from the treatment group mean revertant number), and as mean fold increases compared to the concurrent air control. A ≥2‐fold mean fold increase indicates a positive mutagenic response (as indicatedby the dashed line).

An additional set of EtO spiking experiments were performed in the absence of S9, to determine if the WS matrix had any influence on the mutagenicity of EtO. YG1042 was exposed to three concentrations (460, 2,758, and 5,057 ppm) of EtO in WS using a total diluting flow rate of 3 L/min. This total diluting flow rate was chosen to allow comparison of EtO mutagenicity at a similar concentration range within the WS matrix to the concentration range used to test EtO mutagenicity on its own. The results of these experiments are shown in Figure 7. While there was no evidence of mutagenicity at the top concentration of EtO (5,057 ppm) in one of the experiments, overall the data demonstrate a concentration‐dependent increase in both mean revertant number and fold change compared to the concurrent air control. This was similar to, albeit slightly lower than the response to EtO alone (Figs. 2 and 3), indicating that the presence of WS is unlikely to significantly impact the mutagenicity of EtO in the absence of S9.

## DISCUSSION

The *in vitro* assessment of the toxicity of gaseous compounds has been a challenge for a number of years. This is due to a number of reasons, ranging from safe containment and delivery of the test gas to the cells, to practical considerations surrounding the culturing of bacterial and mammalian cells under conditions that would allow direct exposure to the gas. A number of different exposure systems have been developed and proposed for use in the testing of a wide variety of toxicological endpoints associated with the inhalation of particles, gases and aerosols. The development of these systems has incorporated a multifaceted approach in the design of appropriate exposure chambers, accurate dosing controls and inventive ways of culturing the relevant cellular systems to allow exposure to the test agent at the air–liquid interface [Victorin and Stahlberg, 1988; Aufderheide et al., 2002; Morin et al., 2008; BeruBe et al., 2009; Rach et al., 2013].

In the current study we combined an *in vitro* tobacco smoke exposure system with a gaseous chemical exposure system to allow a more comprehensive assessement of the toxicological activity of gaseous tobacco smoke constituents. To test the gaseous exposure system with a test gas separately before combining it with WS exposure assessment, *Salmonella typhimurium* strain YG1042 cells were treated with EtO for 24 min under conditions that simulated the ISO smoking regime (one 35 mL ‘puff’ of smoke every 60 sec). In our previously described studies on WS the 35 mL puff volume was delivered over an 8 sec exhaust period. For the gas exposures in the present study we have also used a ‘puff’ of gas of 8 sec duration. When combined with a constant gas flow rate of 0.263 L/min, an ISO ‘puff’ volume of 35 mL was achieved. The treatment of YG1042 cells under these conditions with tobacco smoke has previously been shown to induce a mutagenic response [Aufderheide and Gressmann, 2008; Thorne et al., 2013], and we confirmed these findings with our experiments on WS in the current study. For the spiking experiments WS was again generated according to the ISO regime, and test gas (EtO) was added to the WS at various concentrations which were determined by manipulating the gas and diluting air flow rates to vary the ratio of test gas to WS in the mixture that the bacteria were exposed to.

EtO is a tobacco smoke toxicant and was selected for use in this study as it is a direct acting genotoxic agent with known mutagenic properties *in vitro* and *in vivo* [Victorin and Stahlberg, 1988; Agurell et al., 1991; Thier and Bolt, 2000]. A 2‐year exposure to EtO was found to preferentially induce GGT → GTT mutations at codon 12 of K‐*ras* in the lung tumours of B6C3F1 mice [Hong et al., 2007]. As *Ras* mutations are found in approximately 30% of human nonsmall cell lung cancers [Sagawa et al., 1998], this finding may be of relevance to human lung carcinogenesis. The nature of the EtO induced mutations at codon 12 of K‐ras has recently been explored further, and it appears that the selective expansion of mutant clones may play a role in the observed shift in mutational spectrum, rather than this being solely the consequence of a chemically induced genotoxic mechanism [Parsons et al., 2013]. Clearly EtO has a complex mode of action that requires further investigation before it can be fully elucidated.

EtO has previously been shown to be highly mutagenic in *Salmonella typhimurium* strain TA100 when tested at concentrations of 1–200 ppm in a continuous flow system over a 6 hr exposure period, both in the presence and absence of a rat S9 metabolic activation system [Victorin and Stahlberg, 1988]. Further evidence of a mutagenic effect of EtO in the Ames assay was detailed in a study where *Salmonella typhimurium* strains TA100 and TA1535 were exposed to EtO for 4 hr in suspension culture, plated on minimal agar and cultured for 48 hr before scoring of the resulting colonies. EtO was shown to be mutagenic in TA100 and TA1535, inducing a concentration dependent increase in revertant numbers in both bacterial strains [Agurell et al., 1991].

In our study, concentrations of 2697 and 4571 ppm EtO were mutagenic in *Salmonella typhimurium* YG1042 cells, when treated over a 24 min period, simulating the ISO smoking regime. In contrast, cells treated with 995 ppm EtO did not show a statistically significant response. The concentrations of EtO required to elicit a mutagenic response in this assay are relatively high when these are considered in the context of levels of this toxicant in mainstream tobacco smoke. It has been reported that a University of Kentucky 3R4F reference cigarette delivers an average of 9.24 µg EtO over 9 puffs when smoked under ISO conditions [Roemer et al., 2012]. This equates to an average concentration of 16.3 ppm per cigarette in mainstream smoke (based on a 35 mL puff volume), which is much lower than the concentrations of EtO that gave a positive response under ISO conditions in our study. Therefore, albeit based on this rather crude approximation, it appears that EtO may not be a main contributor to the mutagenicity of tobacco smoke. This is further supported by the findings of the WS spiking experiments, where relatively high concentrations of EtO (up to 11,289 ppm in smoke prior to dilution) did not significantly increase the mutagenicity of WS in the presence of S9.

Of the two *in vitro* gaseous exposure studies involving EtO that were discussed earlier [Victorin and Stahlberg, 1988; Agurell et al., 1991], our approach to exposing bacteria to test gas was most similar to the one described by Victorin and Stahlberg, in that the gas was contained within a cylinder and diluted with air prior to exposure of the bacteria. Final concentrations were determined by adjusting the flow rate of the gas, which was limited to three different flow rate settings. However, as only one exposure could be performed per day, each concentration of the test gas was assessed under separate experimental conditions. The authors cite this limitation as a possible cause of significant variation that they reported in their experimental data. Our initial experimental setup also only facilitated the testing of one concentration of EtO at any one time. Therefore, three test runs were required to generate the data for the three concentrations of EtO assessed per experimental dataset. Each of these experimental runs included an air control, and this was used to calculate the mean fold change from control for each test concentration. Later development of the system involved software modifications by the manufacturer and alterations to the setup to enable the control of up to four valves at a time. This allowed all four dilution bars to be utilized to evaluate up to four concentrations of test gas in a single experimental run. When the data generated using the single concentration exposure approach was compared with data obtained from experiments where all three concentrations were tested at the same time, the results were comparable. Our current multiple valve system allows greater potential for increased throughput, and also eliminates a potential source of experimental variation that could arise from the testing of single concentrations of gas individually.

The exposure of bacterial and mammalian cells at the air–agar or air–liquid interface to various test atmospheres such as WS and its gas vapour phase, biological and individual chemical pollutants has previously been described elsewhere [Aufderheide and Gressman, 2007; Pariselli et al., 2009; Schmalz et al., 2011; Wijte et al., 2011; Persoz et al., 2011; Anderson et al., 2013]. These studies have largely been based around the use of either the CULTEX^®^ or Vitrocell^®^ exposure modules.

In this paper we described the use of the Vitrocell^®^ Ames exposure modules for assessment of the *in vitro* mutagenic potential of single gases and spiked WS. We have previously characterized the use of both bacterial and mammalian Vitrocell^®^ exposure modules for testing tobacco smoke, where the effective and reproducible dilution of the smoke by the Vitrocell^®^ system was demonstrated using dosimetry tools [Thorne et al., 2013]. The use of the mammalian modules for testing of gases spiked into WS using the setup described in the present study is therefore possible, and will enable research into the effects of tobacco smoke toxicants on human lung cells, for example. As mentioned earlier, there is a desire amongst national and international health and regulatory authorities to understand the contributions of individual and groups of toxicants to the health risks associated with smoking. WS is a highly complex mixture, and little is still known on the interactions between the individual chemicals within the mixture, and the impact of these interactions on its biological activity. In time, the methodology described here could be further developed to shed more light on this and other aspects of the toxicology of mixtures.

We currently do not have a means of quantifying and therefore confirming the concentration of test gas that the cells are exposed to. Final concentrations are currently calculated based on the dilution of a known concentration of the test gas in the cylinder. The potential addition of gaseous dosimetry tools in the future will enhance our understanding of the dynamics of the system in this regard. The adaptation of the Vitrocell^®^ VC 10 system for combined gaseous and WS exposure as described here offers a great deal of flexibility and control over the concentrations of gas that can be applied, as well as the duration of exposure. The latter is largely limited to the capacity of the gas cylinder and to the length of time that the *in vitro* test system can be cultured at the air–agar or air–liquid interface.

The spiking system described here has broad applicability in many aspects of inhalation toxicology, including investigations into the effects of exposure to environmental pollutants, occupational exposure to gaseous compounds, and studies on the contributions of individual gaseous components to the toxicity of a complex chemical mixture, such as tobacco smoke.

## AUTHORS CONTRIBUTIONS

Damien Breheny prepared the manuscript and provided scientific direction. Fiona Cunningham, Clive Meredith and Debbie Dillon reviewed and provided scientific direction. Joanne Kilford and Rebecca Payne conducted all experiments and provided scientific support. All authors approved the final manuscript.

## References

[em21876-bib-0001] Adamson J , Thorne D , Dalrymple A , Dillon D , Meredith C. 2013 Assessment of cigarette smoke particle deposition within the Vitrocell(R) exposure module using quartz crystal microbalances. Chem Cent J 7:50. 23497606 10.1186/1752-153X-7-50 PMC3635897

[em21876-bib-0002] Agurell E , Cederberg H , Ehrenberg L , Lindahl‐Kiessling K , Rannug U , Tornqvist M. 1991 Genotoxic effects of ethylene oxide and propylene oxide: A comparative study. Mutat Res 250:229–237. 1944340 10.1016/0027-5107(91)90180-v

[em21876-bib-0003] Ames BN , McCann J , Yamasaki E. 1975 Methods for detecting carcinogens and mutagens with the Salmonella/mammalian‐microsome mutagenicity test. Mutat Res 31:347–364. 768755 10.1016/0165-1161(75)90046-1

[em21876-bib-0004] Anderson SE , Khurshid SS , Meade BJ , Lukomska E , Wells JR. 2013 Toxicological analysis of limonene reaction products using an in vitro exposure system. Toxicol In Vitro 27:721–730. 23220291 10.1016/j.tiv.2012.11.017 PMC4680979

[em21876-bib-0005] Araki A , Noguchi T , Kato F , Matsushima T. 1994 Improved method for mutagenicity testing of gaseous compounds by using a gas sampling bag. Mutat Res 307:335–344. 7513814 10.1016/0027-5107(94)90307-7

[em21876-bib-0006] Aufderheide M , Knebel JW , Ritter D. 2002 A method for the in vitro exposure of human cells to environmental and complex gaseous mixtures: application to various types of atmosphere. Altern Lab Anim 30:433–441. 12234248 10.1177/026119290203000406

[em21876-bib-0007] Aufderheide M. 2005 Direct exposure methods for testing native atmospheres. Exp Toxicol Pathol 57:213–226. 10.1016/j.etp.2005.05.019 16092729

[em21876-bib-0008] Aufderheide M , Gressmann H. 2007 A modified Ames assay reveals the mutagenicity of native cigarette mainstream smoke and its gas vapour phase. Exp Toxicol Pathol 58:383–392. 17555953 10.1016/j.etp.2007.02.002

[em21876-bib-0009] Aufderheide M , Gressmann H. 2008 Mutagenicity of native cigarette mainstream smoke and its gas/vapour phase by use of different tester strains and cigarettes in a modified Ames assay. Mutat Res 656:82–87. 18721897 10.1016/j.mrgentox.2008.07.008

[em21876-bib-0010] Bakand S , Winder C , Khalil C , Hayes A. 2005 Toxicity assessment of industrial chemicals and airborne contaminants: Transition from in vivo to in vitro test methods: a review. Inhal Toxicol 17:775–787. 16195213 10.1080/08958370500225240

[em21876-bib-0011] BeruBe K , Aufderheide M , Breheny D , Clothier R , Combes R , Duffin R , Forbes B , Gaca M , Gray A , Hall I , et al. 2009 In vitro models of inhalation toxicity and disease. The report of a FRAME workshop. Altern Lab Anim 37:89–141. 19292579

[em21876-bib-0012] Brody JS , Steiling K. 2011 Interaction of cigarette exposure and airway epithelial cell gene expression. Annu Rev Physiol 73:437–456. 21090967 10.1146/annurev-physiol-012110-142219

[em21876-bib-0013] Burns DM , Dybing E , Gray N , Hecht S , Anderson C , Sanner T , O'Connor R , Djordjevic M , Dresler C , Hainaut P , et al. 2008 Mandated lowering of toxicants in cigarette smoke: A description of the World Health Organization TobReg proposal. Tob Control 17:132–141. 18375736 10.1136/tc.2007.024158 PMC2569138

[em21876-bib-0014] Coggins CR 2010 A further review of inhalation studies with cigarette smoke and lung cancer in experimental animals, including transgenic mice. Inhal Toxicol 22:974–983. 20698816 10.3109/08958378.2010.501831

[em21876-bib-0015] de Serres F , Shelby M. 1979 Recommendations on data production and analysis using the Salmonella/microsome mutagenicity assay. Mutat Res 64:159–165.

[em21876-bib-0016] Doll R , Peto R , Boreham J , Sutherland I. 2004 Mortality in relation to smoking: 50 years' observations on male British doctors. BMJ 328:1519. 15213107 10.1136/bmj.38142.554479.AE PMC437139

[em21876-bib-0017] Food and Drug Administration. Harmful and Potentially Harmful Constituents in Tobacco Products and Tobacco Smoke; Established List. Federal Register 77:2000 34–200037.

[em21876-bib-0018] Hagiwara Y , Watanabe M , Oda Y , Sofuni T , Nohmi T. 1993 Specificity and sensitivity of *Salmonella typhimurium* YG1041 and YG1042 strains possessing elevated levels of both nitroreductase and acetyltransferase activity. Mutat Res 291:171–180. 7685058 10.1016/0165-1161(93)90157-u

[em21876-bib-0019] Hong HH , Houle CD , Ton TV , Sills RC 2007 K‐ras mutations in lung tumors and tumors from other organs are consistent with a common mechanism of ethylene oxide tumorigenesis in the B6C3F1 mouse. Toxicol Pathol 35:81–85. 17325976 10.1080/01926230601063839 PMC2099306

[em21876-bib-0020] Huertas A , Palange P. 2011 COPD: A multifactorial systemic disease. Ther Adv Respir Dis 5:217–224. 21429981 10.1177/1753465811400490

[em21876-bib-0021] IARC . 2008 IARC monographs on the evaluation of carcinogenic risks to humans. Volume 97. 1,3‐butadiene, ethylene oxide and vinyl halides (vinyl fluoride, vinyl chloride and vinyl bromide). IARC Monogr Eval Carcinog Risks Hum 97:3–471. PMC4782275 20232717

[em21876-bib-0022] Kilford J , Thorne D , Payne R , Dalrymple A , Clements J , Meredith C , Dillon, D . 2014 A methodology for the assessment of mainstream cigarette smoke genotoxicity using the bacterial reverse mutation assay and an aerosol‐based exposure system. Mutat Res (in press). 10.1016/j.mrgentox.2014.04.017 25344108

[em21876-bib-0023] Maron DM , Ames BN. 1983 Revised methods for the Salmonella mutagenicity test. Mutat Res 113:173–215. 6341825 10.1016/0165-1161(83)90010-9

[em21876-bib-0024] Morin JP , Hasson V , Fall M , Papaioanou E , Preterre D , Gouriou F , Keravec V , Konstandopoulos A , Dionnet F. 2008 Prevalidation of in vitro continuous flow exposure systems as alternatives to in vivo inhalation safety evaluation experimentations: Outcome from MAAPHRI‐PCRD5 research program. Exp Toxicol Pathol 60:195–205. 18472257 10.1016/j.etp.2008.01.007

[em21876-bib-0025] Owen K. 2013 Regulatory toxicology considerations for the development of inhaled pharmaceuticals. Drug Chem Toxicol 36:109–118. 22273711 10.3109/01480545.2011.648327

[em21876-bib-0026] Pariselli F , Sacco MG , Rembges D. 2009 An optimized method for in vitro exposure of human derived lung cells to volatile chemicals. Exp Toxicol Pathol 61:33–39. 18650076 10.1016/j.etp.2008.05.008

[em21876-bib-0027] Parsons BL , Manjanatha MG , Myers MB , McKim KL , Shelton SD , Wang Y , Gollapudi BB , Moore NP , Haber LT , Moore MM. 2013 Temporal changes in K‐ras mutant fraction in lung tissue of big blue B6C3F1 mice exposed to ethylene oxide. Toxicol Sci 136:26–38. 24029818 10.1093/toxsci/kft190

[em21876-bib-0028] Persoz C , Leleu C , Achard S , Fasseu M , Menotti J , Meneceur P , Momas I , Derouin F , Seta N. 2011 Sequential air–liquid exposure of human respiratory cells to chemical and biological pollutants. Toxicol Lett 207:53–59. 21840384 10.1016/j.toxlet.2011.07.028

[em21876-bib-0029] Rach J , Budde J , Mohle N , Aufderheide M. 2013 Direct exposure at the air–liquid interface: Evaluation of an in vitro approach for simulating inhalation of airborne substances. J Appl Toxicol 5:506–515. 10.1002/jat.2899 23765558

[em21876-bib-0030] Rodgman A , Perfetti TA . 2013 The Chemical Components of Tobacco and Tobacco Smoke, 2nd ed Boca Raton: CRC Press p 2332.

[em21876-bib-0031] Roemer E , Schramke H , Weiler H , Buettner A , Kausche S , Weber S , Berges A , Stueber M , Muench M , Trelles‐Sticken E , et al. 2012 Mainstream smoke chemistry and in vitro and in vivo toxicity of the reference cigarettes 3R4F and 2R4F. Beiträge zur Tabakforschung International/Contributions to Tobacco Research 25:316–335.

[em21876-bib-0032] Sagawa M , Saito Y , Fujimura S , Linnoila, RI. 1998 K‐ras point mutation occurs in the early stage of carcinogenesis in lung cancer. Br J Cancer 77:720–723. 9514049 10.1038/bjc.1998.118 PMC2149957

[em21876-bib-0033] Schmalz C , Wunderlich HG , Heinze R , Frimmel FH , Zweiner C , Grummt T. 2011 Application of an optimized system for the well‐defined exposure of human lung cells to trichloramine and indoor pool air. J Water Health 9:586–596. 21976205 10.2166/wh.2011.144

[em21876-bib-0034] Thier R , Bolt HM. 2000 Carcinogenicity and genotoxicity of ethylene oxide: New aspects and recent advances. Crit Rev Toxicol 30:595–608. 11055837 10.1080/10408440008951121

[em21876-bib-0035] Thorne D , Adamson J. 2013 A review of in vitro cigarette smoke exposure systems. Exp Toxicol Pathol 65:1183–1193. 23850067 10.1016/j.etp.2013.06.001

[em21876-bib-0036] Thorne D , Kilford J , Payne R , Adamson J , Scott K , Dalrymple A , Meredith C , Dillon D. 2013 Characterisation of a Vitrocell(R) VC 10 in vitro smoke exposure system using dose tools and biological analysis. Chem Cent J 7:146. 24004496 10.1186/1752-153X-7-146 PMC3844484

[em21876-bib-0037] Victorin K , Stahlberg M. 1988 A method for studying the mutagenicity of some gaseous compounds in *Salmonella typhimurium* . Environ Mol Mutagen 11:65–77. 3276508 10.1002/em.2850110108

[em21876-bib-0038] Wijte D , Alblas MJ , Noort D , Langenberg JP , van Helden HP. 2011 Toxic effects following phosgene exposure of human epithelial lung cells in vitro using a CULTEX^®^ system. Toxicol In Vitro 25:2080–2087. 21945045 10.1016/j.tiv.2011.09.003

[em21876-bib-0039] Zhong BZ , Gu ZW , Whong WZ , Wallace WE , Ong TM. 1991 Comparative‐study of micronucleus assay and chromosomal aberration analysis in V79 cells exposed to ethylene‐oxide. Teratog Carcinog Mutagen 11:227–233. 1687900 10.1002/tcm.1770110502

